# Education Research: Integration of Trainee and Faculty Clinics at an Academic Medical Center: Improving Quality of Care and Education

**DOI:** 10.1212/NE9.0000000000200328

**Published:** 2026-06-12

**Authors:** Matthew Schelke, Anna C. Cai, Comana Cioroiu, Dina Dababneh, Mihaela Negrutiu, Nasly C. Montealegre, Jennifer Perez-Caro, Michelle Bell, Inna Kleyman, Kirk Roberts, Richard Mayeux, Olajide A. Williams

**Affiliations:** 1 Department of Neurology and the Neurological Institute of New York, Columbia University, NY; and; 2 New York Presbyterian Hospital, NY.

## Abstract

**Background and Objectives:**

Trainees provide a large proportion of neurologic care at academic medical centers in the United States. However, compared with their corresponding faculty practices, traditional trainee practices are often physically separate, serve primarily government-insured patients, and provide poorer continuity-of-care and reduced access to subspecialty services. We attempted to mitigate these challenges by merging the trainee and faculty practices at the Columbia University Department of Neurology over the 2022–2023 academic years.

**Methods:**

Over the 2022–2023 academic years, the Department merged the neurology trainee and faculty practices by embedding trainees fully within faculty clinics. We describe our integration process and share preliminary data on its effects on continuity of care and access to subspecialty services. We also share results from preintegration and postintegration surveys to residents and faculty demonstrating improvement in education and mentorship experience.

**Results:**

By the 2023 academic year, trainees were fully embedded in faculty clinics and paired with longitudinal faculty mentors. This improved continuity of care for the patients in the former trainee clinics from 87.3% to 99.6% (*p* < 0.001) and a trend toward increase of subspecialty referrals from 41.5% to 45.5% (*p* = 0.142). It also significantly improved the educational and mentorship experience for residents and faculty.

**Discussion:**

This study shows that integration of trainee and faculty practices is feasible and can improve continuity of care for patient groups who often receive inequitable neurologic care in academic centers. It can also significantly enhance the educational and mentorship experience for both trainees and faculty.

## Introduction

Neurology trainees provide a substantial portion of outpatient clinical care at academic medical centers (AMCs) in the United States. Typically, neurology trainees rotate through a resident or fellow continuity clinic, in which they see patients with a wide variety of neurologic illnesses and learn how to care for these patients under the aegis of faculty preceptors. This system is designed to allow trainees to develop longitudinal relationships with patients and receive clinical mentorship from faculty members.

In practice, however, the physical separation of trainee and faculty practices based on health insurance status often leads diminished continuity of care with providers. This is linked to the finite 3-year period residents spend in the clinic before graduating from the residency program and the rotating faculty preceptor model. Because outpatient physician continuity of care has been associated with reduced emergency room utilization, hospitalization, lower costs, and greater patient satisfaction,^1^ eliminating this structural inequity was considered a primary goal of the integration process and help address the perception that the existing academic practice model is separate and unequal.

Along with disrupted continuity of care, quality of care and educational experience is diminished in typical resident clinics by additional factors. First, residents often review their cases (staff the cases) with different faculty mentors over their training, which exposes them to different practice styles but reduces mentorship.^2,3^ Second, the volume of patient visits often limits time for teaching and discussion.^3,4^ In many clinics, attendings will discuss cases rather than seeing and examining resident patients themselves and teaching at bedside. Together, these both reduce the ability of attendings to provide longitudinal care for patients over time and to provide adequate supervision and mentorship to their trainees.

To address the inequity, some AMCs have restructured the trainee clinics, reducing the ratio of residents to faculty preceptors,^5^ creating interprofessional teams to enhance continuity of care and improve access to core resources,^6^ and separating clinic rotations from inpatient rotations to reduce conflicts between the demands of these settings.^7^ Others have engaged the community directly in developing clinical and population health interventions, like Chicago's Community Grand Rounds,^8^ or involved community members and patients in the governing bodies of residency clinics, similar to patient participation in community health center administrations.^9^ Although all these changes are important concrete steps, they do not address the separate and unequal concerns: the framework of physically separating clinic populations by insurance type (Medicaid vs commercial). US history has shown that systems that separate people based on their financial ability, or its surrogates, often end up segregating by race/ethnicity, which has been shown to perpetuate unconscious biases and has been linked to disparate care. Furthermore, physically separating clinics based on insurance type also generates 2 different clinical experiences from the perspective of the trainee, supervising attending, and patient and has led to ongoing concerns across neurology residency practices that outpatient resident training is inadequate for both trainees and patients.^10,11^

The Department of Neurology Columbia University Irving Medical Center in partnership with NY-Presbyterian Hospital (NYPH) and Columbia Doctors attempted to address this inequity by fully integrating our trainee and faculty practices during the 2022–2023 academic years. Before this integration, the department had physically separate trainee and faculty clinics in which the former saw patients primarily insured by Medicaid and the latter primarily saw patients insured by Medicare or commercial insurance (henceforth M/CI). Patients in the trainee clinic had poorer continuity of care, and we hypothesized that integrating the 2 practices into a single, payer-agnostic model would improve continuity of care. A secondary hypothesis was that the payer-agnostic model would mitigate the observed reduced referrals to subspecialty care in the trainee clinic compared with patients with similar neurologic conditions in the faculty practice (FP). Finally, we hypothesized that the clinic merger would improve the resident and faculty educational experience including their perceived continuity of care and opportunities for feedback and mentorship.

Our operational process and preliminary clinical-related outcomes are described in this report.

## Methods

Before the 2022–2023 academic years, our Department of Neurology operated separate trainee and faculty clinics. The resident clinic (RC), operated by NYPH, was the primary outpatient site for all residents and fellows and served primarily patients with Medicare and Medicaid insurance, along with a minority of private health insurance plans. The FP, operated by ColumbiaDoctors, included the clinical practices of most of the faculty members and served primarily patients with Medicare and private insurance plans.

We established a Task Force comprising departmental clinical operation administrators, finance leaders, select neurology division chiefs, the diversity, equity, and inclusion leader, residency program directors, and chief residents. The Task Force was charged with expanding the neurology ambulatory practice with the goal of providing: (1) the best care to all patients, regardless of ability to pay; (2) the best educational experience to our residents; and (3) examining clinic models from peer institutions for adaptable best practices. Key governing principles included: (1) all clinical faculty (general and subspecialists) would be required to participate and see all insurances regardless if they are working with trainees; (2) the new integrated model would be budget neutral; (3) the health insurance plans accepted by providers would be homogenized across the department to facilitate payer agnostic care and equity of access; and (4) the integrated model complied with the Graduate Medical Education requirements regarding resident education.

We also established a Community Advisory Committee (CAC) who reviewed our proposed integrated model framework and made additional recommendations. The 7 members of the CAC included 3 diverse neurology patients, 1 Chief Executive Officer of a local Federally Qualified Health Center, 1 community social worker, 1 community health worker, and 1 certified health insurance enrollment officer. Recommendations from the CAC included increasing social work and translation services, equity and cultural competency training for all providers and staff, and having a clear process for communicating the clinic transition process to patients, including those who may have low literacy levels.

Recommendations from the Task Force and CAC were implemented by the Department of Neurology with support from NYPH and Columbia Doctors. The partnership between New NYPH and the Department was crucial to support the clinic integration, and the financial resources targeted for the original RC in the Hospital were reassigned to the Department to support the merged practice. The study, a quality improvement project, received Institutional Review Board (IRB) exemption. There was no outside funding source for this research.

### Impact Assessment Variables

To assess the preliminary impact of the integration on patient care, we extracted electronic medical record (EMR) data for the 8-month period before the merger from September 1 to June 1, 2021–2022, and for the same date ranges following the merger in 2023–2024. Primary comparisons included assessing changes in specific quality of care metrics extracted from the EMR among Medicaid vs MC/I patients seen in the RC and FP before and after the merger. These EMR data included: (1) continuity of care: proportion of patients seeing the same resident/attending team across at least 2 visits; (2) referral to specialty care assessed by the proportion of patients referred to subspecialists; and (3) evaluating the effect of payer agnostic triaging at the call center level by measuring wait time for new patient appointments by insurance type. Statistical analyses for the comparisons were performed using 2-tailed Z tests with correction for multiple comparisons. Patient demographics were collected through the available EMR data.

### Educational Experience Assessment

To assess the educational impact of this restructuring, we distributed surveys to residents and general neurology attendings 1 month before the merger of the neurology practices (the presurvey) and 6 months after the merger (the postsurvey). We also collected EMR data on multiple metrics, including patient payer mix and continuity of care. Questions included in the resident survey targeted domains identified in the studies described above as most important for the educational experience of a continuity clinic: longitudinal relationship with patients, mentorship and teaching by attendings, and comfort managing common outpatient neurologic diagnoses (eTable 1). Questions included in the attending survey targeted similar domains, including opportunities for teaching, relationship with patients, and ability to provide feedback to residents (eTable 2). We also sent a single postintegration survey assessing the resident's comfort managing typical outpatient issues in their 2 neurologic subspecialties. Most response options were formulated as three-element or four-element Likert scales.

### Standard Protocol Approvals, Registrations, and Patient Consents

This project was reviewed by the Columbia University IRB and deemed exempt from IRB approval as it involved quality improvement data which were collected as part of routine patient care. Participant consent was not required.

### Data Availability

Anonymized data not published within this article will be made available by request from any qualified investigator.

## Results

The Division of Epilepsy FP was the first to integrate the RC in June 2022 when it began accepting all health insurance plans. Over the subsequent 15 months, each RC—epilepsy, neuromuscular medicine, movement disorders, aging and dementia, stroke, and finally, general neurology—were consecutively integrated into the FP. This process was completed by September 2023.

### Clinic Schedule Optimization

Before the clinic integration, residents rotated in the RC for 1 week every 6 weeks, participating in 5–6 clinic sessions (half-days) every week. These half-days were a mixture of general neurology and specialty sessions (with different specialty sessions for the resident during each rotation week) and were supervised by a rotating group of attendings who each would supervise 3–5 residents per session. New patient visits were allotted 60 minutes and follow-up patients 30 minutes. There was no specific built-in time for attending precepting.

After the integration, residents rotate in the FP for 1 week every 4 weeks, joining 2 general neurology sessions and 2 subspecialty sessions (split between 2 different subspecialties) during each week. In contrast to the prior structure, attendings work with a single resident per session and see their own patients in parallel. Unlike the RC model in which residents had back-to-back schedules with ad hoc precepting time, the integrated clinics included specific built-in staffing time for the attending to review the case with the resident and for both to see the patient together. In addition, the rotating faculty preceptor system was replaced with a system that shifted the overarching responsibility for continuity of care from residents (who graduate and leave) to attending faculty. In the postintegration model, residents are assigned a single general neurology attending for the entire 3 years of their residency, and each year they are assigned 2 separate subspecialty attendings. This allows them to experience 6 different subspecialties over the 3 years of neurology training.

Columbia neurologists are compensated through a relative value unit (RVU) system, which requires the neurologist to meet specific RVU goals. As all RVUs from the resident sessions accrue to the supervising attending, the impact on their RVUs was neutral to net positive (every session converted to a resident session had space for at least 1 or 2 more patients than the equivalent solo attending session). Previously, attendings volunteered their time at the NYPH RC without direct insurance reimbursements accruing to the Department. The goal of budget neutrality for the Department was achieved both through funding from NYPH for the care of patients previously seen in the RC and through the reimbursements that now accrue to the Department as attendings are billing under the Department instead of under NYPH.

### Social Work and Translation Services

Additional social workers were hired into the FP, and an outpatient social work team was created with a dedicated departmental leader to support multiple divisions. This new social worker team launched new patient and family centered initiatives to support vulnerable patients including a new monthly caregivers orientation program for addressing issues such as advanced care planning, Medicaid benefits, homecare options, workplace accommodations, respite, Supplemental Security Income and Social Security Disability Insurance, Social Determinant of Health barriers such as transportation to appointments, and other general topics submitted by caregivers and care teams daily. We also expanded our language service capacity with the addition of interpreters with rolling video carts (“Interpreter on Wheels”) and created new opportunities for attendings and residents to learn medical Spanish via an Ambulatory Care Network class.

### Data Analysis

In the preintegration period, we examined data on 5,512 patient visits to neurology: 792 (14.4%) of these patients had Medicaid as their primary insurance and 4,720 (85.6%) had Medicare or commercial insurance (M/CI). In the postintegration period, we examined data on 5,830 patient visits to neurology: 589 (10.1%) had Medicaid, and 5,241 (89.9%) had M/CI. Although, consistent with established data highlighting the different sociodemographic profiles of Medicaid vs commercially insured patients, sociodemographic data, while different across the patient groups (Medicaid and M/CI patients), remained consistent over the duration of the integration period (Table 1). Medicaid patients were younger, more likely to be Black and Hispanic, and more likely to live in an urban zip code compared with M/CI patients (all *p* < 0.001).

**Table 1:** Demographic Comparison of Our Study Population

	Medicaid in RC	Medicaid in IP	M/CI in FP	M/CI in IP
Age, y mean ± SD	45 ± 15	44 ± 14	55 ± 18	57 ± 18
Sex, % female	72	74	66	67
Race				
% White	13	17	57	51
% Black	19	19	9	12
% other (inc. Hispanic)	54	48	11	20
Location, % urban	94	89	51	61

FP = faculty practice from September 2021 through June 2022; IP = integrated practice from September 2023 through June 2024; M/CI = Medicare and commercial insurance; RC = resident clinic from September 2021 through June 2022.

### Continuity of Care

**Figure 1:**
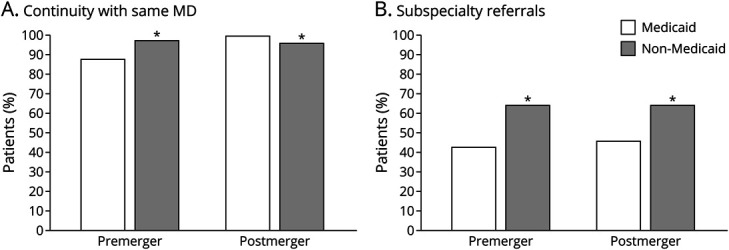
Continuity of Care and Subspecialty Referrals Proportion of patients with Medicaid vs non-Medicaid insurance who had continuity with the same neurologist (panel A) and were referred to a subspecialty practice (panel B) before and after the clinic merger. In Panel A, the proportion of Medicaid patients who saw the same neurologist before the integration was significantly lower than that of non-Medicaid patients (87.3% vs 97.2%, *p* < 0.001); after the integration, it was slightly but significantly higher than that of non-Medicaid patients (99.6% vs 95.6%, *p* = 0.0027). The difference between the preintergration and postintegration Medicaid proportions was also significant (*p* < 0.001). In Panel B, the proportion of Medicaid patients referred to a neurologic subspecialist before the integration was significantly lower than that of non-Medicaid patients (41.5% vs 64.1%, *p* < 0.001), and this disparity persisted after the integration (45.5% vs 63.8%, *p* < 0.001). Statistical comparisons were performed using a 2-proportion Z test. * = statistically significant value.

Before the integration, the proportion of RC (Medicaid) patients who saw the same medical doctor across visits (87.3%) was lower than that of FP (M/CI) patients (97.2%), with a statistically significant difference between the 2 groups (*p* < 0.001, Figure 1). After the integration, the Medicaid group had a significantly higher proportion of patients who saw the same medical doctor, 99.6%, compared with 95.6% of non-Medicaid patients (*p* = 0.003, Figure 1A). The increase in continuity for the Medicaid patients (87.3%–99.6%) was statistically significant (*p* < 0.001) (Figure 1A).

### Subspecialty Referrals

Before the integration, 41.5% of RC patients were referred to subspecialists compared with 64.1% of patients referred from FP visits (*p* < 0.001, Figure 1). After the integration, there was no significant change in the difference between the 2 groups, with MC/I patients remaining significantly more likely to be referred to subspecialists than Medicaid patients (*p* < 0.001, Figure 1B). However, a slight nonsignificant increase in specialty visit referrals was observed for Medicaid patients after integration from 41.5% to 45.5% (*p* = 0.142, Figure 1B).

### Payer Agnostic Triage

Although our overall wait times increased following the merging of both clinics, the difference in wait time for new patient visits between Medicaid and commercially insured patients decreased after the merger to meet our predetermined goal of not more than 5 days. Before the merger, the difference in wait time for new patient appointments between Medicaid and commercially insured patients was 13 days (45 vs 32 days). After the merger, this difference was 5 days (96 vs 91 days). As new patients were assigned to available slots (either trainee-attending pairs or solo attendings) regardless of their insurance status, there was no significant difference in the proportion of patients cared for by trainee-attending pairs across insurance groups after the merger (2% Medicare, 4% Medicaid, 1% commercial insurance, not significant).

### Educational Assessment

Of 30 adult neurology residents, 17 (56.6%) responded to the presurvey and 18 (60%) to the postsurvey. Respondents were evenly distributed among the 3 postgraduate years. Of 8 general neurology attendings, 8 (100%) responded to the presurvey and 6 (75%) to the postsurvey.

**Figure 2:**
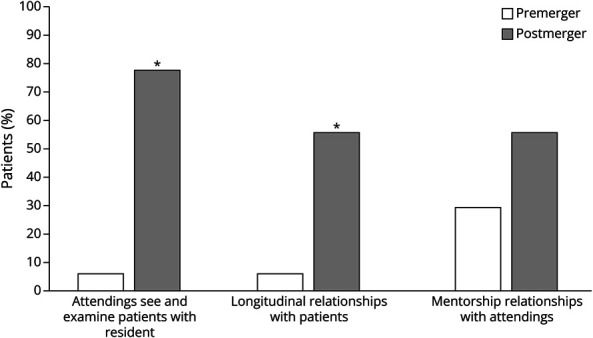
Feedback From the Educational Survey to Residents The proportion of residents selecting the highest response category increased from 6% to 78% for attendings who see and examine every patient in clinic (*p* < 0.00001), increased from 6% to 56% for having longitudinal relationships with all or almost all patients (*p* = 0.0016), and did not change for mentorship relationships with all outpatient attendings (29%–56%, *p* = 0.142). eTable 1 for survey. Statistical comparisons were performed using a 2-proportion Z test. Premerger, n = 18 respondents; postmerger, n = 17 respondents. * = statistically significant value.

**Figure 3:**
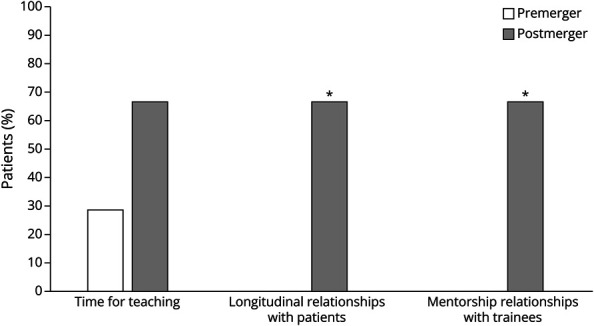
Feedback From the Educational Survey to Attendings The proportion of attendings selecting the highest response category did not change for very adequate time for teaching (29% vs 67%, *p* = 0.171), increased from 0% to 67% for having longitudinal relationship with all or almost all patients (*p* = 0.0093), and increased from 0% to 67% for having a mentorship relationship with all or almost all of the residents they work with (*p* = 0.0093). eTable 2 for survey. Statistical comparisons were performed using a 2-proportion Z test. Premerger, n = 8 respondents; postmerger, n = 6 respondents. * = statistically significant value.

Both resident and attending responses revealed significant enhancement of the educational experience in the new clinic structure. The frequency with which attendings saw and examined patients with the residents and the proportion of patients with whom residents felt they had a longitudinal relationship both increased significantly, while the frequency of perceived attending mentorship showed a trend toward increase but did not reach significance (Figure 2). From the attending perspective, the ability to mentor and provide feedback to residents and longitudinal relationships with patients also all increased significantly, whereas the adequacy of teaching time showed a trend toward improvement but did not reach significance (Figure 3). Samples of qualitative feedback supported these perceived improvements in quality of educational experience (Table 2).

**Table 2:** Representative Feedback; Quotations Taken Directly From Feedback Forms Without Modification

Role	Feedback
Resident	“I enjoyed the more consistent continuity with patients and how much more streamlined it felt connecting our patients to other services and obtaining important diagnostic tests”
Resident	“Being able to work one-on-one with an attending has given me the opportunity to dive deeper on each patient, learning more details about the science but also more of the art of medical decision-making.”
Attending	“With the merger, I am able to gain a deeper understanding of each resident in my resident pod. I know their strengths and areas for development and am able to provide targeted clinical mentorship.”

## Discussion

In this study, we described the process of integrating the physically separate resident practices which served Medicaid patients, and faculty practices which served Medicare and commercially insured patients, into a single payer-agnostic system of outpatient neurologic care to reduce operational inequities.

Our preliminary data show that before the merger, there were baseline disparities between the practices in continuity of care and referrals to subspecialty care. The RC had less continuity and referrals to specialty care compared with the FP. Eight months after the merger, we saw notable improvements in continuity of care but not referrals to subspecialty care.

Improvement in continuity of care was likely due to the revised scheduling system in which residents were paired with longitudinal mentor attendings for the 3 years of neurology residency. This meant that patients followed by the same resident were guaranteed to follow with the same attending, and if resident appointments were not available (for urgent visits during a specific week, for example), the patients were seen by the attending rather than assigned to a new resident.

Although we did not find significant improvement regarding referral to subspecialty care before and after the merger across both Medicaid and Medicare/commercially insured groups, we did find persistent disparities in referral to subspecialty care, which was significantly lower among Medicaid beneficiaries compared with Medicare/commercial insurance. Interestingly, although nonsignificant, we observed improvement in subspecialty care referrals among Medicaid beneficiaries but not for Medicare/commercial insurance beneficiaries after the merger. The persistent disparity observed regarding referral patterns in our payer-agnostic homogenized insurance acceptance model may be related to our observation that half of the M/CI patients were from outside the city, and although not specifically analyzed, we have found that these patients usually seek second or third opinions that require a higher rate of subspecialty referral compared with Medicaid patients who are mostly local and often present for neurologic conditions that can be adequately addressed in a general neurology clinic. We do not have current data on the diagnosis distribution between these groups, but this is an area for future research.

One major finding was a significant increase in wait times for new patients—rising from 45 to 96 days for Medicaid patients and from 32 to 91 days for commercially insured patients. A key driver of this increase was a sharp rise in referral volume as more primary care clinics and hospitals across the New York City area became aware of our new payer-agnostic triage process. This surge in demand coincided with changes to our supervision model, compounding the impact on appointment availability. Previously, attendings supervised 3–4 residents per session without dedicated staffing time and did not see every patient directly. Under the new structure, attendings were required to see every patient and were paired 1:1 with residents, greatly reducing capacity. To address these constraints, we have begun testing several strategies, including reducing staffing time and shifting some sessions to a 2:1 supervision model while still ensuring that all patients are seen by both residents and attendings.

From the educational survey, we found that the clinic merger enhanced the educational experience for both residents and attendings. Attendings saw and examined many more patients at bedside with the resident (a feature that was built into the clinic structure) and attendings felt that they had more time for teaching, although the latter finding did not reach significance likely because of the small sample size. Both residents and attendings felt that they had longitudinal relationships with a larger proportion of patients and mentorship relationships with each other, although the perceived mentorship from the resident survey did not reach significance. This latter finding may be because the survey was sent 6 months after the merger and thus did not reflect the full length of the attending-resident pairings. The subjective comments supported these findings. Importantly, although not all residents completed the survey, a majority did; as it was anonymous we cannot identify whether the nonresponders were the same for both the presurveys and postsurveys which does open the possibility of nonresponse bias affecting the results.

Study limitations: Our findings are limited by several factors. First, the experience at Columbia may not be generalizable to all institutions. Columbia has a relatively large general neurology group who all practice at least part of the week at the main hospital campus. Programs with mostly subspecialists or with physicians dispersed among multiple locations may not be able to reproduce our methodology exactly. Second, the study duration of 8 months many have been an insufficient period for observing meaningful changes in referral patterns. Third, we did not specifically track new patients entering our system between the observed periods, which may have introduced measurement errors related differences in the premerger and postmerger patient populations, although we did find similar sociodemographic characteristics premerger and postmerger. Fourth, we note that following the merger, we experienced higher no show rates in the merged practice, largely driven by Medicaid beneficiaries, which has been reported extensively in the literature.^12,13^ Both in the literature and in our experience, no-show rates in the Medicaid population are driven by multiple systemic factors including unreliable transportation (both public transit and medical transportation arranged through Medicaid), limitations in home aide hours provided by Medicaid, and inconsistent access to appointment reminder modalities (email and telephone service, for example).

Finally, as with any multicomponent intervention, it is challenging to isolate which elements were most impactful. Nonetheless, educational feedback suggests that guaranteed continuity of care between patients and a consistent attending, along with the redesigned scheduling templates, were among the most valuable improvements for both quality of care and the resident learning experience. At the same time, these changes introduced several unintended consequences. Fixed trainee–attending pairings may have created disparities in educational exposure and limited residents' access to diverse teaching and practice styles. In addition, the increased rigidity of the scheduling templates reduced the clinic's flexibility to accommodate urgent visits, which may have further contributed to longer wait times.

We have already implemented corrective measures, including the launch of a designated rapid-access clinic in which residents work with attendings outside of their usual pairings to see urgent outpatient referrals.

In this report, we describe and present preliminary data on the integration of resident and faculty practices into a single payer-agnostic FP model facilitated by the homogenization of health insurance plans. Our results suggest that merging the resident and faculty practices into a single system of neurologic outpatient care can significantly improve continuity of care, which has been linked to reduced emergency room utilization, hospitalization, lower costs, and greater patient satisfaction.^1^ Such a merger can also significantly improve the educational experience for both trainees and attendings, strengthening relationships with patients and mentorship relationships within the practice.

## Glossary

AMC: academic medical center
CAC: community advisory committee
EMR: electronic medical record
FP: faculty practice
IRB: institutional review board
NYPH: NY-Presbyterian Hospital
RC: resident clinic
RVU: relative value unit
